# The Proteasome Inhibitor Bortezomib Enhances ATRA-Induced Differentiation of Neuroblastoma Cells via the JNK Mitogen-Activated Protein Kinase Pathway

**DOI:** 10.1371/journal.pone.0027298

**Published:** 2011-11-07

**Authors:** Peihua Luo, Meili Lin, Lin Li, Bo Yang, Qiaojun He

**Affiliations:** College of Pharmaceutical Sciences, Institute of Pharmacology and Toxicology, Zhejiang University, Hangzhou, China; Dana-Farber Cancer Institute, United States of America

## Abstract

Neuroblastoma (NB) is the most common extracranial solid tumor in childhood. Differentiated human NBs are associated with better outcome and lower stage; induction of differentiation is considered to be therapeutically advantageous. All-trans retinoic acid (ATRA) has been shown to induce the differentiation of neuroblastoma (NB) cell lines. The proteasome inhibitor bortezomib inhibits cell growth and angiogenesis in NBs. Here, we investigated the synergistic effect between bortezomib and ATRA in inducing NB cell differentiation in different NB cell lines. Bortezomib combined with ATRA had a significantly enhanced antiproliferative effect. This inhibition was characterized by a synergistic increase in neuronal differentiation. At the same time, the combination therapy showed little neuronal toxicity which was assessed in primary cultures of rat cerebellar granule cells by the MTT assay, PI staining. The combination of bortezomib and ATRA triggered increased differentiation through the activation of proteins, including RARα, RARβ, RARγ, p-JNK and p21, compared with ATRA treatment alone. Using JNK inhibitor SP600125 to block JNK-dependent activity, the combination therapy-induced neuronal differentiation was partially attenuated. In addition, p21 shRNA had no effect on the combination therapy-induced neuronal differentiation. The in vivo antitumor activities were examined in human NB cell xenografts and GFP-labeled human NB cell xenografts. Treatment of human NB cell CHP126-bearing nude mice with ATRA plus bortezomib resulted in more significant tumor growth inhibition than mice treated with either drug alone. These findings provide the rationale for the development of a new therapeutic strategy for NB based on the pharmacological combination of ATRA and bortezomib.

## Introduction

Therapeutic approaches based on the induced differentiation of transformed cells into mature cells are one of the most promising strategies in recent NB treatments [1]. Retinoids represent the most frequently used group of differentiation inducers, which are used both in leukemias and in some types of solid tumors, like neuroblastoma (NB) [2]–[3]. However, evidence of potential toxicity and intrinsic or acquired resistance substantially limits the use of retinoids in clinical protocols. Special attention has thus been paid to the combined treatment of retinoids and other compounds that enhance or modulate the effect of retinoids on differentiation [4]. As previous reports have shown, all-trans retinoic acid (ATRA)-induced cell differentiation in the SH-SY5Y NB cell line can be enhanced by combined treatment with either interferon α2a [5] or inhibitors of LOX/COX [6].

NB originates from sympathetic neuroblasts of the peripheral nervous system and is one of the most common solid childhood tumors that accounts for 7–10% of childhood cancers and around 15% of childhood cancer deaths [7]. Despite the current advances in treatment, the clinical prognosis of aggressive NB remains dismal. For these reasons, combination chemotherapy currently represents one of the major successes in oncology research, due to its acceptable systemic toxicity and appreciable efficacy [8]. In addition, because NBs are classified as embryonal tumors that arise from immature cells of the neural crest, induced differentiation of NB cells has become a currently used therapeutic protocol. As such, differentiation therapy with retinoids is of special interest in current studies [9]–[10]. Rather than focusing on cytotoxicity, we focused on NB differentiation. Prior clinical data had suggested that differentiation therapy might play a complementary role in the treatment of NB when used in combination with other therapies.

Previous studies indicated that RARα expression was ubiquitin-dependent and decreased during ATRA-induced neuronal differentiation. The proteasome inhibitor MG132 can increase cellular sensitivity to retinoic acid through the inhibition of RARα catabolism and amplification of RARα transcription. Furthermore, ATRA treatment might induce cell differentiation in NB cells that are normally insensitive to retinoic acid (RA) [11]–[12]. Bortezomib, which is also known as PS-341 or Velcade, is a potent and selective inhibitor of the 26 S proteasome that is currently being evaluated for the treatment of various cancers [13]. It has also received Food and Drug Administration approval for the treatment of multiple myeloma [14]. Due to its serious adverse effects on the nervous system in patients, researchers have redesigned the drug formulation to reduce its toxicity [15].

Based on these reports, we hypothesized that bortezomib could enhance ATRA-induced differentiation. In the present study, we investigated the potential synergistic effect of bortezomib in combination with ATRA in vitro and in vivo and the associated mechanism. Because NB is a neuron-based cancer and bortezomib is toxic to neurons, we tested whether the combination therapy would affect the neuronal toxicity of bortezomib. Our primary goal was to provide guidelines for the design of clinical test models for combination treatments with bortezomib and ATRA.

## Materials and Methods

This study was carried out in accordance with the National Institute of Health Guide for the Care and Use of Laboratory Animals. The protocol was approved by the Committee on the Ethics of Animal Experiments of the Zhejiang University (Permit Number: Zju2009101004 and Zju2010101033).

### Reagents

ATRA was from Sigma Chemical Co. (Sigma-Aldrich,USA). Bortezomib was gifts from Topharman (Topharman, Shanghai, China) for research use only, and dissolved in DMSO (1.0 mM stock solution) and stored at −20°C.

The primary antibodies to β-actin, Tau, β tubulin III, RARα, RARβ, p-ERK p-JNK, p-p38, p21, caspase 3, Bax, Bcl-2 and HRP-labeled secondary anti-goat, anti-mouse, and anti-rabbit antibodies were all purchased from Santa Cruz Biotechnology (Santa Cruz, USA). ECL was purchased from Amersham Biosciences (Piscataway, NJ). 3-(4,5)-dimethylthiahiazo (-z-y1)−3,5-diphenytetrazoliumromide (MTT) was purchased from Sigma (Sigma-Aldrich, USA). Propidium iodide (PI) and JNK inhibitor SP600125 were both purchased from BD (Biosciences Pharmingen, USA).

### Cell culture

Human CHP126 cells were gifts from University of South California, and SH- SY5Y were purchased from the Cell Bank of the China Science Academy (Shanghai, China). These cells were cultured in RPMI1640 (Invitrogen, USA) supplemented with 10% fetal bovine serum(FBS) (Invitrogen, USA) containing 100 U/mL penicillin and 100 µg/mL streptomycin (Sigma-Aldrich, USA), and incubated at 37°C in a humidified 5% CO_2_ atmosphere. For green fluorescent protein (GFP) gene transduction, CHP126 cells were stable transfected into the pZsGreen-1 Vector (Clontech, USA) by Lipofectamine™ 2000 (Invitrogen, USA). Transfected CHP126 cells (CHP126-GFP cells) were cultured into a selective medium that contained 800 µg/mL of G418 (Geneticin, Roche, Germany) for 7 days. The brightest fluorescing cells above the 90 percentile were sorted and cloned. Transfected cells were maintained by selection with 400 µg/mL of G418.

### Cell Growth Curve

NB cells were plated into 24-well plates, twenty-four hours after plating, cells were exposed with DMSO (0.01%), ATRA (10.0 nM), bortezomib (5.0 nM) or as described. At different time points, the cells were detached with trypsin solution and counted with a hematocytometer, using a trypan blue solution. The experiments were repeated three times with three replicates each [16].

### Morphometric Analysis

SH-SY5Y and CHP126 cells were grown on 6 well plates treated with 10 nmol/L ATRA or vehicle alone (0.01% DMSO) in DMEM/Ham's F-12 containing 10% fetal bovine serum followed by incubation at 37°C for 3 days. Neuronal differentiation of NB cells was observed with phase-contrast microscopy, with two pathologists identifying the morphologic changes to neuronal differentiation. For quantification, cells with neurites longer than 50 µm were counted as neurite positive under microscope. At least 100 cells were counted per sample. All experiments were done in triplicate and the results presented as means with SE [17].

### Immunofluorescence assay

SH-SY5Y cells were grown on 96 wells plate and incubated in DMEM/Ham's F–12 containing 10% fetal bovine serum followed by the treatment as described for 5 days. Cells were then fixed by 4% paraformaldehyde in PBS at room temperature for 60 min and permeabilized by 10% formaldehyde in PBS containing 0.1% Triton X-100 at room temperature for 10 min. Detergent-permeabilized cells were blocked with PBS/2% bovine serum albumin at room temperature for at least 1 h and were incubated with a goat anti-tau or β-tubulin III antibody in PBS/0.1% bovine serum albumin at 4°C overnight. Following extensive washes with PBS, cells were incubated with an Alexa Fluor 488 donkey anti-goat IgG in PBS/0.1% bovine serum albumin at room temperature for 1 h. Immunofluorescence images were obtained by using a Leica florescence microscope. The fluorescence intensity of tau and β-tubulin III was quantitated by Leica Application Suite Advanced Fluorescence and analyzed by Student's t test. Data were shown as the mean ± SE of the average fluorescence per cell from five different viewing areas [18].

### Realtime PCR

Total RNA from CHP126 and SY-SH5Y cells was isolated using the Trizol reagent (Sangon Biotech Co., Ltd), and cDNA was synthesized using 2 µg of total RNA with random hexamer primers and the Moloney murine leukemia virus reverse transcriptase (M-MuLV RT) (Fermentas International Inc., Burlington, Ontario, Canada). The conditions used for reverse transcription-PCR were as follows: 10 min at 25°C, 60 min at 42°C and 15 min at 72°C. The cDNA was subjected to PCR amplification using the following forward and reverse primer sets: RET, forward primer: 5′- TGTGGAGACCCAAGACATCA-3′ and reverse primer: 5′- CCGAGACGATGAAGGAGAAG-3′; GAP43, forward primer: 5′- GGAGAAGGCACCACTACTGC-3′ and reverse primer: 5′- GGCGAGTTATCAGTGGAAGC-3′; TrkB, forward primer: 5′- TGTGGAGACCCAAGACATCA-3′ and reverse primer: 5′- CCGAGACGATGAAGGAGAAG-3′; GAPDH, forward primer: 5′-GTCATCCATGACAACTTTGG-3′ and reverse primer: 5′-GAGCTTGA CAAAGTGGTCGT-3′. The housekeeping gene GAPDH was used as the internal standard. PCR products were separated on 1.0% agarose gel and visualized by ethidium bromide staining. Gels were photographed using a Gel DOC 2000 image analyzer (Bio-Rad, Hercules, CA, USA). The quantitative real-time RT-PCR analysis was performed by TAKARA SYBR Premix EXTaqTM. The reaction mixtures containing SYBR Green were composed following the manufacturer's protocol. The cycling program was 95°C for 30 s, 58°C or 70°C (GADD153) for 20 s, and 72°C for 30 s followed by 40 cycles using an Eppendorf epGradient Mastercycler (Eppendorf, Hamburg, Germany).

### Generate p21 knockdown CHP126 cells

Stable p21 knockdown CHP126 cells and were generated by transfection of plasmids. Briefly, cells were seeded into six-well plates at approximately 60–70% confluence 12–24 h before transfections, next day were stable transfected into the p21 shRNA (Santa Cruz, USA) by Lipofectamine™ 2000 (Invitrogen, USA) according to the manufacturer's instructions. Cell monolayers were trypsinized 24 h after transfection and transferred into T25 flasks or 100-mm diameter culture dishes. Cells were then selected by growth in complete medium containing 2 µg /mL of puromycin for 4 weeks. Viable clones were pooled together and cultured for expansion in T75 Flasks and at the same time assayed by western blot to ensure p21 knockdowns [19].

### The isolation and Cell Culture of Primary rat cerebellar granule neuron

Primary cultures of cerebellar granule neurons were prepared as follows. The cerebellar region was removed from postnatal 2nd day Wistar rat under a stereoscopic microscope. The tissue was placed in Ca2+- and Mg2+-free Dulbecco's phosphate buffer saline (CMF-DPBS, pH 7.4) containing 3 g/L BSA (Sigma-Aldrich, USA), 1 g/L glucose (Sigma-Aldrich, USA), and 50 µg/mL gentamicin (Gibco, USA). The meninges were removed from the cerebella tissue, which was then placed on sterile Teflon board. A sterile scalpel was used to slice the sample using perpendicular strokes before it was transferred to a 50 mL collection tube. It was then briefly centrifuged, and the pellet was resuspended in 12 mL of a warm solution of 0.025% trypsin (Gibco. USA) and 0.04% DNAse (Sigma-Aldrich, USA). The suspension was incubated in a shaking water bath for 10 min, followed by addition of 1 mL of fetal calf serum (FCS) to inhibit trypsin activity and 1 mL of 1 mg/mL DNAse to decrease the clumping of genomic DNA from lysed cells. The suspension was then centrifuged at 800 rpm for 5 min. After removing the supernatant, the pellet was resuspended and slowly triturated with a fire-polished glass pipette in Dulbecco's Modified Eagle's Medium (DMEM, pH 7.4) (Gibco, USA) containing 19 mM NaHCO3, 26.2 mM KCl, 7 µM p-aminobenzoic acid (Sigma-Aldrich, USA), 100 mU/L insulin (Sigma-Aldrich, USA), 50 µg/mL gentamicin, and 10% FCS. The cells were plated at a density of 1.5×10^6^ per ml in 96-well plates, 10 cm Petri dishes and 8-well chamber slides. All culture surfaces were pretreated with poly-Lysine. Cultures were maintained in 5% CO2/95% air at 37°C. Experiments were carried out on the third to fifth day in vitro. Immunocytochemical analysis showed that 90% of cells were neurons (data not shown) [20].

### Neuron cell viability assay

Primary neurons cells were treated with various concentrations of bortezomib and ATRA alone or combined for 72 hours. Cells were then incubated with MTT (5.0 mg/mL, 20 µL/well). After 4 h, the culture medium was removed, and the formazan granules generated by live cells were dissolved in DMSO. The absorbance was measured at 570 nm using a multiskan spectrum (Thermo Electron Co., Finland). The inhibition rate on cell proliferation was calculated for each well as (A570 control cells - A570 treated cells)/A570 control cells×100% [21].

### PI staining for flow cytometry

The sub-G1 analysis after PI staining was employed to assess the apoptosis. Primary neuron cells (5×10^5^/well) were seeded into 6-well plates and exposed to bortezomib, ATRA or bortezomib combined with ATRA for 4 days. Cells were then harvested and washed with PBS, fixed with pre-cooled 70% ethanol at 4°C overnight. Cell pellets were resuspended in 1 mL of 0.1% sodium citrate containing propidium iodide (PI) 0.01 mg and 50.0 µg RNase for 30 min at room temperature in the dark. For each sample at least 5×10^4^ cells should be analyzed using an FACS-Calibur cytometer (Becton Dickinson, USA) [22].

### Western blot

Cells (5×10^5^/well) were treated with compounds for 24 h. Proteins were extracted with lysis buffer (50 mM Tris–HCl, 150 mM Nacl, 1 mM EDTA, 0.1% SDS, 0.5% deoxycholic acid, 0.02% sodium azide, 1% NP-40, 2.0 µg/ml aprotinin, 1 mM phenylmethylsulfonylfluoride). The lysates were centrifuged at 10,000 g for 30 min at 4°C. Equivalent amounts of proteins were analyzed by SDS–PAGE. Appropriate antibodies to: anti-RARα, anti-RARβ, anti-RARγ, anti-ERK, anti-JNK, anti-p38, anti-pERK, anti-pJNK, anti-pp38, anti-β-actin, anti-Tau, anti-β tubulin III, anti-p21, anti-caspase 3, anti-Bax and anti-Bcl-2 from Santa Cruz Biotech (Santa Cruz, USA). Proteins were visualized with HRP-labeling secondary antibody, using ECL-plus kit for detection [23].

### GFP-labeling human neuroblastoma cell xenografts

Female Balb/C nude mice (National Rodent Laboratory Animal Resource, Shanghai, China), 4–5 weeks of age, were used for all experiments. For surgical procedures, mice were anaesthetized using ketamine 50 mg/kg and xylazine 35 mg/kg intraperitoneal injection (i.p.).Implantation was performed through a midline incision practiced under the microscope. A total of 5×10^6^ GFP-labeling CHP126 cells in 15 µL DMEM were injected in the left hippocampus using a 33 G needle connected to a Hamilton syringe. After recovery, the animals were kept in a germ-free protected area, with food adlibitum. Treatment started 3 days after tumor cell injection (day 0) and mice were then randomly assigned to four groups and treated with bortezomib and ATRA administered individually or in combination, or with saline solution (control mice). Bortezomib (0.5 mg/kg) and ATRA (5.0 mg/kg) were intravenous injection (i.v.) or intragingival injection (i.g.) injected twice a week for a total of 4 wk, with a 3-d interval between injections. Body weight and general physical status of the animals were recorded daily until mice were sacrificed by cervical dislocation after being anesthetized with xilezine. A Leica fluorescence stereo microscope model MZ FL III (Leica, Germany) were used for photographing and accounting the GFP-expression area respectively. Selective excitation of GFP was produced through a D470/40 band-pass filter and 510 DCXR dichroic mirrors. Emitted fluorescence was collected through a longpass filter DC300FX and digital camera system (Leica, Germany). The areas of neuroblastoma xenografts on the brain were counted under the fluorescence stereo microscope. GFP fluorescence area and total brain area were measured by using Image-Pro plus 5.0 software (MediaCybernetics, USA). The GFP-expressing area on the brains was measured to quantify the tumor growth. Animal studies were preformed in accordance with protocols approved by the ZJU Animal Subjects Committee.

### Human neuroblastoma cell xenografts

Female Balb/C nude mice (National Rodent Laboratory Animal Resource, Shanghai, China), 4–5 weeks of age, were used for all experiments. Human neuroblastoma cell CHP126 xenografts were established by 5×10^6^ cells subcutaneously inoculated in nude mice. Treatments were initiated when tumors reached a mean group size of 100 mm3. The mice were randomized to no treatment (control), Bortezomib (0.5 mg/kg, i.p. administration), ATRA (5.0 mg/kg, i.g. administration), or the combination of Bortezomib plus ATRA twice a week for 20 days. The size of tumors were measured individually twice per week withmicrocalipers. Tumor volume (V) was calculated as V = (length×width)^2^/2. The individual relative tumor volume (RTV) was calculated as follows: RTV = Vt/V0, where Vt is the volume on each day of measurement and V0 is the volume on the day of initial treatment. Therapeutic effect of compound was expressed in terms of T/C% and the calculation formula is T/C (%) = mean RTV of the treated group/mean RTV of the control group×100%. The tumor growth inhibition rate was calculated using the formula IR (%) = (1 − TWt/TWc)×100, where TWt and TWc are the mean tumor weight of treated and control groups, respectively. Animal studies were preformed in accordance with protocols approved by the ZJU Animal Subjects Committee.

### Statistical Analysis

Data were expressed as the mean ± SE from at least three separate experiments, and statistical significance was assessed by Student's two-tailed unpaired t-test. (**p < 0.01 and ***p<0.001.)

## Results

### Bortezomib enhances ATRA-inhibited **cell proliferation** in SH-SY5Y and CHP126 cells

**Figure 1:**
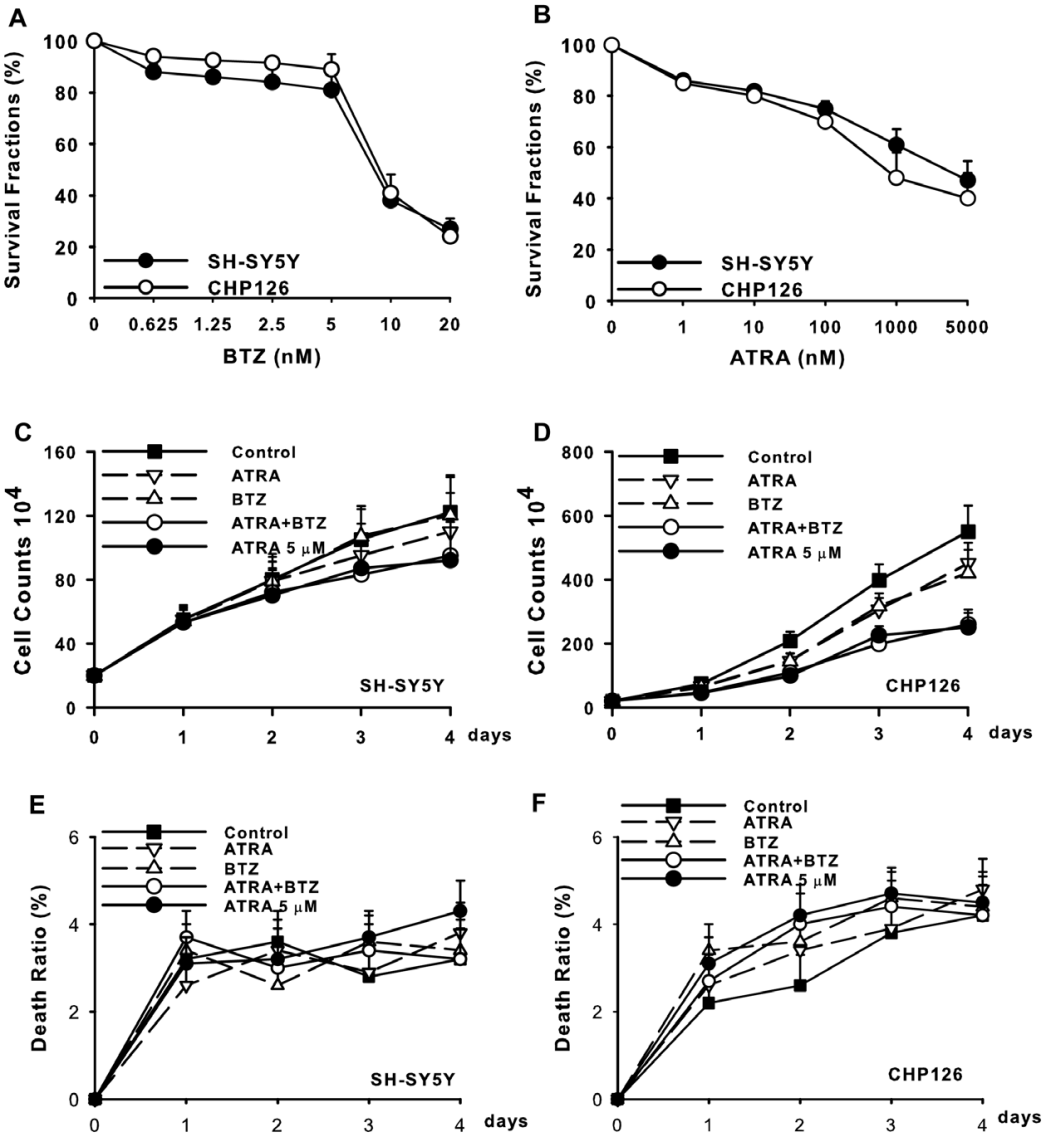
Bortezomib promoted ATRA-induced growth arrest in human neuroblastoma CHP126 and SH-SY5Y cells. SH-SY5Y and CHP126 cells were seeded and treated with bortezomib (A) or ATRA (B) for 96 h, and Trypan blue staining was used to detect the surviving cells. Dose-response curves of the two cell lines treated with bortezomib (A) and ATRA (B) are presented. SH-SY5Y (C) and CHP126 (D) cells were treated as described above. The cell growth curves were created by counting cells each day. Massive cell death of SH-SY5Y and CHP126 cells was induced by treatments (E and F). Data are shown as the means ± SE (n = 3).BTZ  =  bortezomib.

The neuroblastoma cell lines SH-SY5Y and CHP126 were tested for sensitivity to bortezomib and ATRA in vitro by Trypan blue staining. As shown in Fig. 1A and B, bortezomib and ATRA caused a dose-dependent decrease in cell viability. The concentrations of bortezomib and ATRA used for differentiation therapy were determined according to the growth inhibition rates of bortezomib (5.0 nM) and ATRA (10.0 nM) in both cell lines; moreover, they did not induce cell death. Therefore, we investigated the effect of bortezomib on ATRA-inhibited cell proliferation by treating SH-SY5Y and CHP126 cells with 10.0 nM ATRA in the absence or presence of 5 nM bortezomib. ATRA (5.0 µM) used alone significantly induced a cell growth arrest up to 4 days after treatment as shown in Fig. 1C and D. A marked enhancement in the inhibition of cell proliferation was observed when 5.0 nM bortezomib was added to cells in the presence of ATRA (10.0 nM), which was similar to the result obtained with 5.0 µM ATRA alone (Fig. 1C and D). In addition, the combination of ATRA and bortezomib only weakly induced cell death (Fig.1E and F).

### Bortezomib synergistically increases ATRA-induced **cell differentiation** in SH-SY5Y and CHP126 cells

**Figure 2:**
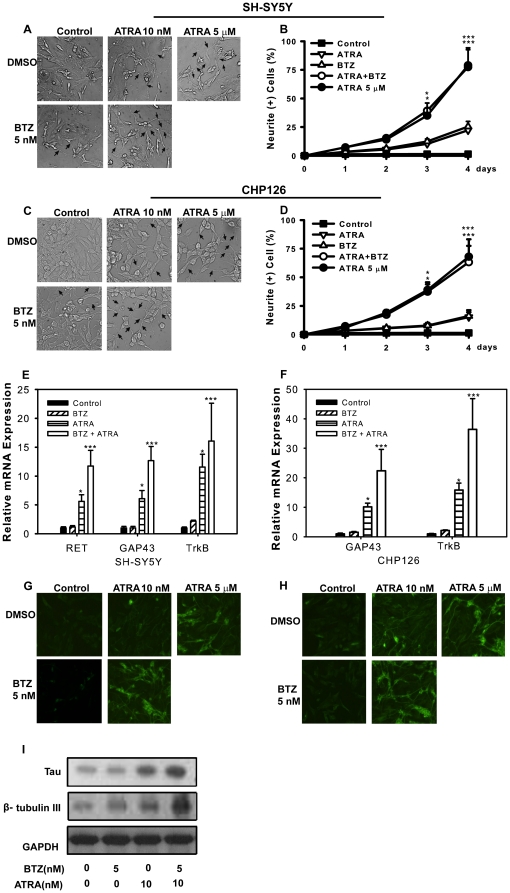
Bortezomib synergistically increases ATRA-induced cell differentiation in SH-SY5Y and CHP126 cells. SH-SY5Y (A) and CHP126 (C) cells were treated with 5.0 nM bortezomib, 10.0 nM ATRA and 5.0 nM bortezomib plus 10.0 nM ATRA, 5.0 µM ATRA, as well as under control conditions. The morphology of the cells was observed by microscopy at the same magnification. The microphotographs shown are representative of three different experiments. The images were analyzed with the Leica software to determine the relative lengths of the neurites (B and D). Data are shown as the means ± SE (n = 3). SH-SY5Y and CHP126 cells were incubated as above, and realtime-PCR was performed to detect the expression of RET, GAP43 and TrkB (E and F). SH-SY5Y cells were incubated as above, and immunofluorescence was performed to detect the expression of tau and β tubulin III (G and H). Western blotting (I) was used to detect the expression of tau and β tubulin III in CHP126 cells. BTZ = bortezomib.

To determine whether the bortezomib-mediated growth inhibition was accompanied by differentiation, we determined the extent of differentiation using standard assays for neurite maturation. The phenotypic characteristics of NB cell lines were evaluated by microscopic inspection of the overall morphology after 4 days of treatment. Treatment with ATRA (5.0 µM) resulted in significant differentiation and the appearance of a neuronal phenotype and produced more adherent cells that are spread out and polar and had longer neurites that were interconnected with frequent varicosities (Fig. 2A and C). About 70% of the cells exhibited processes neurites longer than 50 µm, which agrees with previously reported results [24] for these cells (Fig. 2B and D). ATRA (10.0 nM) and bortezomib (5.0 nM) treatment alone induced fewer morphological changes, which gave rise to cells that sprouted numerous short neuritic processes with occasional varicosities (Fig. 2A and C). The combination of both drugs had dramatic effects on the SH-SY5Y and CHP126 cell morphologies; about 70% of the cells exhibited processes longer than 50 µM, which is similar to the results obtained with ATRA alone (5.0 µM, Fig. 2B and D).

To determine whether the growth arrest and morphological changes induced by the combination treatment of ATRA and bortezomib were accompanied by enhanced differentiation in NB cells, the expression of differentiation markers was examined. Real-time PCR was applied on SH-SY5Y and CHP126 cells to detect differentiation markers (RET, GAP43 and TrkB). For CHP126 doesn't express endogenous RET, we only detected GAP43 and TrkB expression. It was indicated that mRNA levels of all neuronal markers were further increased by bortezomib in presence of ATRA in both SH-SY5Y and CHP126 cells(Fig. 2E and F).Immunofluorescence staining was performed on SH-SY5Y cells exposed to ATRA (10.0 nM) and bortezomib (5.0 nM) alone or together using antibodies against β tubulin III and tau. β tubulin III is a marker of neuronal maturation, while tau is used as a marker of mature neurons(24). Ten nanomolar ATRA alone induced a moderate increase in β tubulin III and tau expression levels, whereas bortezomib (5.0 nM) did not cause any change. When 5 nM bortezomib was administered with 10 nM ATRA, the levels of both β tubulin III and tau increased dramatically and were observed in almost all cells (Fig. 2G and H). Western blot was applied on CHP126 cells to detect the expression of β tubulin III and tau. Both were obviously upregulated with combined treatment, while just slightly increased with bortezomib or ATRA alone treatment (Fig. 2I).

### Protein expression levels of RARα, RARβ, RARγ, p21, JNK, pJNK, ERK, pERK, p38 and pp38 during ATRA and bortezomib-induced cell differentiation

**Figure 3:**
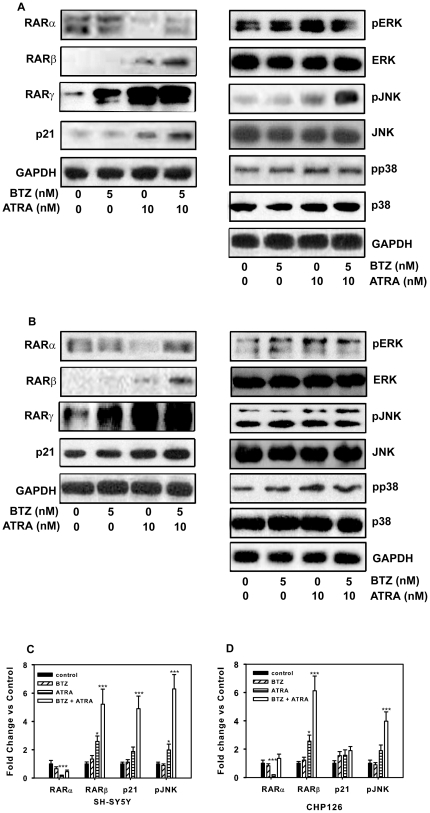
Protein expression levels in ATRA and bortezomib-induced differentiation. SH-SY5Y (A) and CHP126 (B) cells were incubated with 5 nM bortezomib, 10 nM ATRA and bortezomib plus ATRA, as well as under control conditions for 72 h. Western blotting (WB) was used to detect the expression of RARα, RARβ, RARγ, p21, JNK, pJNK, ERK, pERK, p38 and pp38. Densitometric analysis of the autoradiographic plaques of significantly changed proteins in SH-SY5Y (C) and CHP126 (D) cells. Statistical analysis was performed by t-test where appropriate. *P<0.05, **P<0.01,***P<0.001, significant difference from control cells. BTZ = bortezomib.

To obtain insight into the signaling pathway involved in the neuronal differentiation amplification activity of bortezomib, western blotting analyses of proteins that have been reported to be potential differentiation mediators were performed. Total cell extracts were obtained from SH-SY5Y cells (Fig. 3A) and CHP126 cells (Fig. 3B) after treatment with ATRA or bortezomib for 4 days. Previous reports showed the crucial involvement of retinoic acid receptors (RARs) α and β in ATRA-induced NB cell differentiation [25]; therefore, we examined the expression levels of the two RAR isoforms in the NB cell lines treated with ATRA and bortezomib. Several studies demonstrated that RARα mediates the differentiation of some types of cancer cell lines. Our study indicated that ATRA downregulated RARα expression, while bortezomib (5.0 nM) blocked the ATRA-mediated RARα reduction in both NB cell lines (Fig. 3A and B, left panel). RARβ is an ATRA-inducible tumor suppressor that plays an important role in neuroblastoma cell differentiation. In agreement with previous reports [26] that ATRA can increase RARβ expression, we found that treatment with ATRA (10.0 nM) increased RARβ expression, and its expression level was further increased in the presence of bortezomib (Fig. 3A and B, left panel). Previous reports indicated that increased RARγ expression suppresses the malignant phenotype and alters the differentiation potential of human neuroblastoma cells[27], here we detected ATRA or bortezomib alone can upregulate RARγ expression, while no further increase with combined treatment.

**Figure 4:**
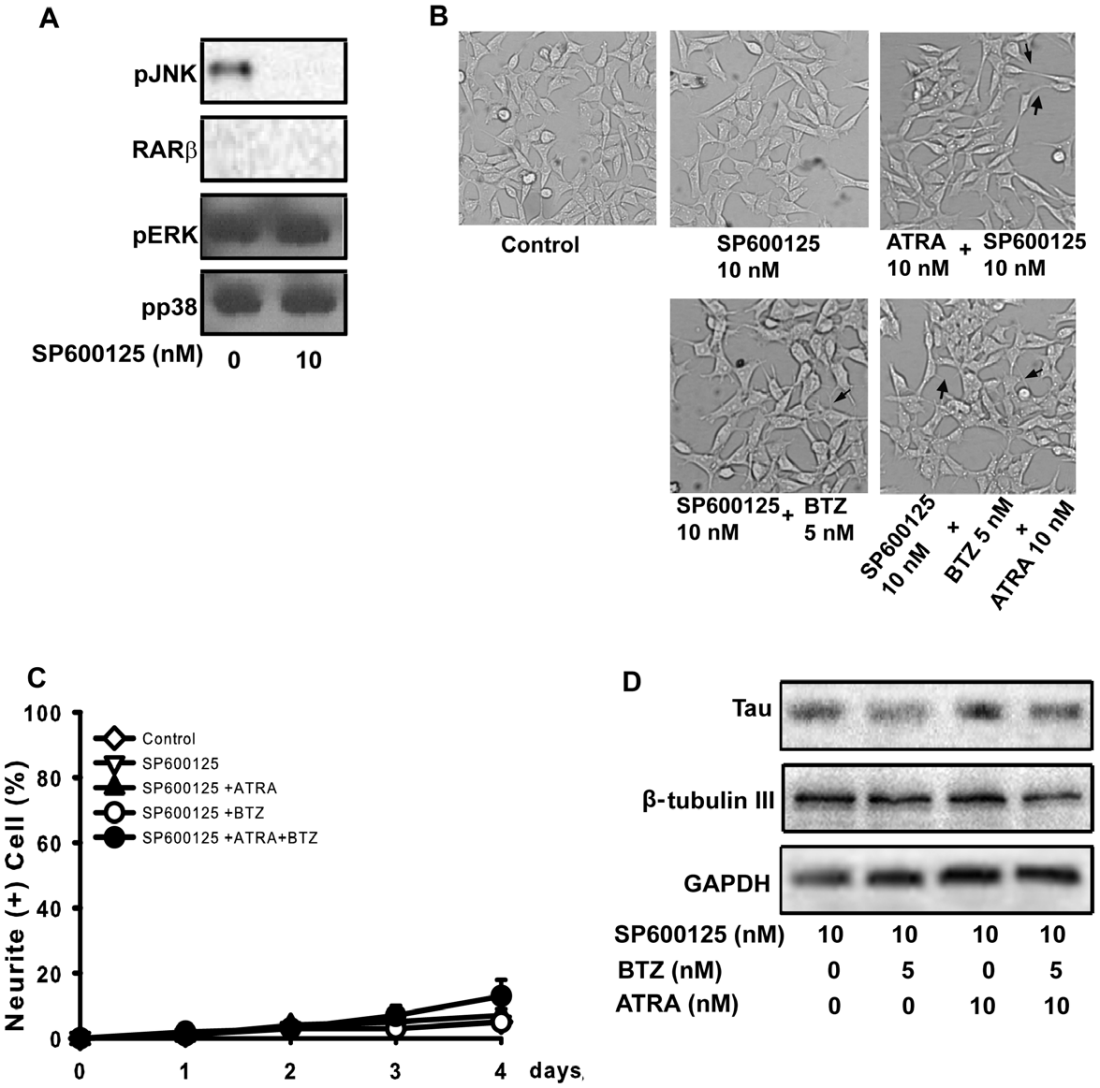
JNK is involved in the enhancement of ATRA-induced differentiation of neuroblastoma cells by bortezomib. A: WB was used to detect the expression of pJNK, RARα, RARβ, pERK and pp38 after SP600125 treatment. B: CHP126 cells were treated with 5.0 nM bortezomib, 10.0 nM ATRA and 5.0 nM bortezomib plus 10.0 nM ATRA in the presence of SP600125, as well as under control conditions. The morphology of cells was observed by microscopy at the same magnification. The microphotographs shown are representative of three different experiments. The images were analyzed with the Leica software to determine the relative lengths of the neurites (C). Data are shown as the means ± SE (n = 3). D. WB was used to detect the expression of tau and β tubulin III with or without SP600125 treatment. BTZ = bortezomib.

Recent studies suggested that the MAPK pathway plays a pivotal role in neuronal differentiation [28]. To determine whether the MAPK pathway is involved in the combined treatment-induced neuronal differentiation of SH-SY5Y and CHP126 cells, the expression levels of MAPK pathway components (pERK, pJNK and pp38) were determined by western blotting. As shown in the right panel of Fig. 4A and B, the combination of bortezomib and ATRA did not cause a further increase in pERK and pp38 expression, but the upregulation of the pJNK level by bortezomib was further increased in the presence of ATRA (Fig. 3A and B). These results suggest that the regulation of JNK plays a more important role than other MAPK components in the repression of neuroblastoma cell differentiation in the combined treatment.

p21/Waf1 expression has been closely correlated with induced differentiation in multiple cell culture model systems, including human neuroblastoma cells [29]. In our study, the combination of bortezomib with 10.0 nM ATRA caused a further upregulation of the p21Waf1/Cip1 levels (Fig. 3A and B, left panel), suggesting that the regulation of p21 also plays an important role in the repression of NB cell differentiation with the combined treatment.

### JNK is involved in the enhancement of ATRA-induced differentiation of neuroblastoma cells by bortezomib

To elucidate the role of JNK in ATRA-enhanced neuronal differentiation induced by bortezomib, CHP126 cells were treated with ATRA or bortezomib alone or both drugs together in the presence or absence of the JNK inhibitor SP600125. The concentration of SP600125 used was based on a previous study in which it failed to cause morphological change or death in CHP126 cells (data not shown). The combined therapy related pathway remained same after this concentration of SP600125 treatment (shown in Fig. 4A). The morphological changes and neuronal marker expression patterns with various treatments were observed. Four days after bortezomib and ATRA treatment in the presence of SP600125, neuronal differentiation was not observed (Fig. 4B). In contrast, bortezomib synergistically increased ATRA-induced CHP126 cell morphological changes in the absence of the JNK inhibitor (Fig. 2A). The numbers of CHP126 cells with processes longer than 50 µm were different among the various treatments. SP600125 reduced the cell numbers that were induced by bortezomib and ATRA alone or in combination (Fig. 4C). Therefore, bortezomib and ATRA-induced neurite outgrowth was blocked by treatment with the JNK inhibitor. To further examine whether JNK mediates neuronal marker expression, we treated cells with bortezomib and ATRA in the presence or absence the JNK inhibitor and assayed for the expression of tau andβ tubulin III by western blotting. The combined treatment-induced expression of tau andβ tubulin III was inhibited by the JNK inhibitor (Fig. 4D), indicating that JNK is necessary for bortezomib-enhanced neuronal differentiation induced by ATRA.

### Inhibition of p21 expression has no effect on bortezomib and ATRA-induced differentiation of CHP126 cells

**Figure 5:**
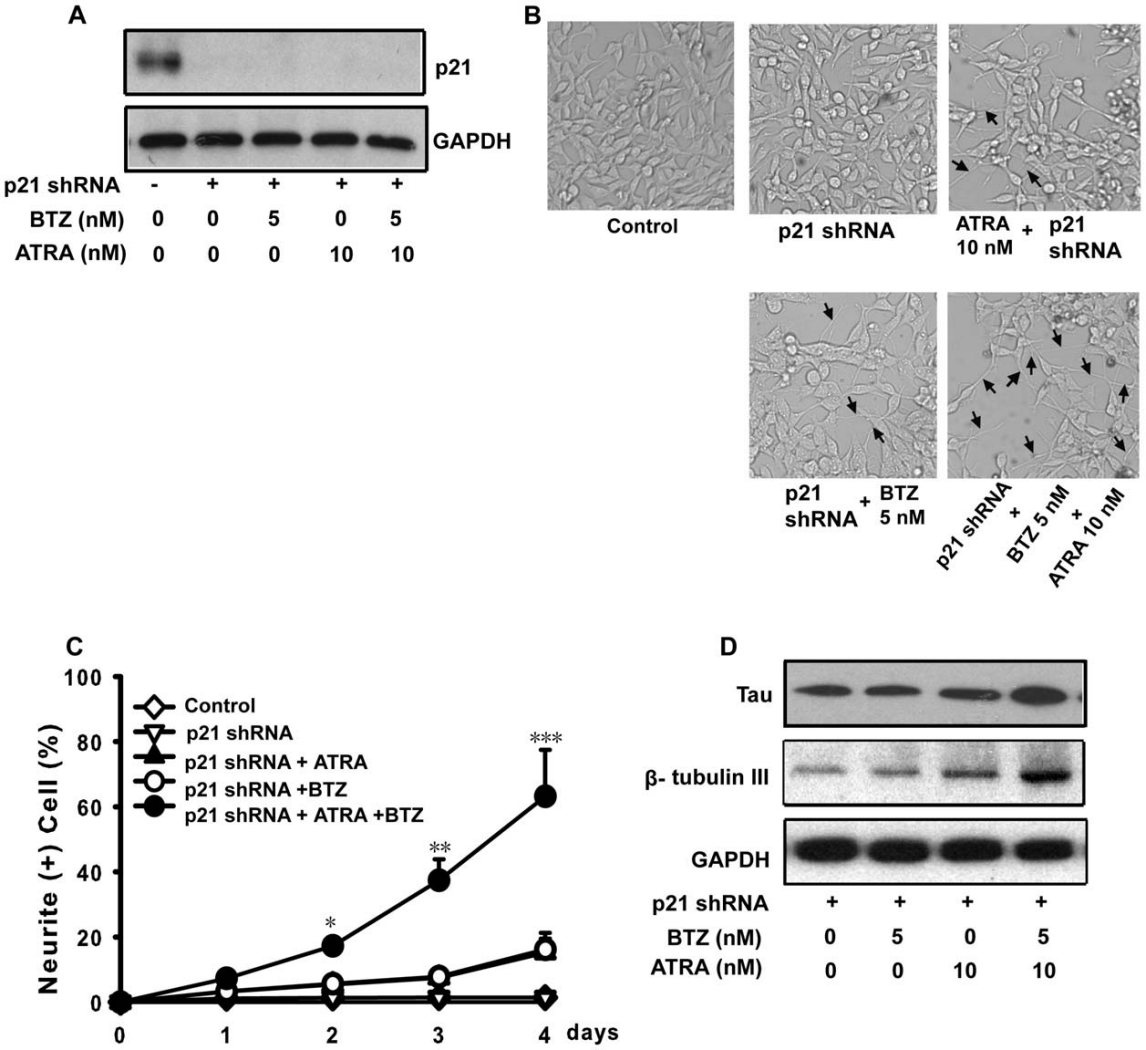
p21 is not involved in the enhancement of ATRA-induced differentiation of neuroblastoma cells by bortezomib. A: p21 expression was detected in Stable p21 knockdown or control CHP126 cells. B: Stable p21 knockdown or control CHP126 cells were treated with 5.0 nM bortezomib, 10.0 nM ATRA and 5.0 nM bortezomib plus 10.0 nM ATRA. The morphology of cells was observed by microscopy at the same magnification. C: The microphotographs shown are representative of three different experiments. The images were analyzed using the Leica software to determine the relative lengths of the neurites. Data are shown as the means ± SE (n = 3). D. WB was used to detect the expression of tau and β tubulin III. Statistical analysis was performed by t-test where appropriate. *P<0.05, **P<0.01,***P<0.001, significant difference from control cells. BTZ = bortezomib.

Because p21 was strongly increased in both NB cells treated with a combination of bortezomib and ATRA, we then determined whether blocking p21 expression resulted in the inhibition of NB cell differentiation. We introduced p21 shRNA to generate stable p21 knockdown cells (as shown in Fig. 5A). The cell number did not differ between the controls or p21 knockdown cells treated with bortezomib and ATRA (data not shown). Therefore, p21 depletion did not inhibit NB proliferation. As shown in Fig. 2A and 5B, bortezomib and ATRA treatment did not produce any morphological differences between the controls and p21 knockdown cells. The numbers of NB cells exhibiting processes longer than 50 µm were almost the same in the various treatments (Fig. 5C). In our experiment, p21 is effectively reduced by shRNA even when cells are treated with both BTZ and ATRA (Fig. 5C). The expression levels of neuroblastoma differentiation markers did not change following treatment, as analyzed by western blotting (Fig. 5D). All together, inhibition of p21 expression did not directly affect bortezomib-enhanced ATRA-induced neuronal differentiation of neuroblastoma cells.

### Combined antitumor activity of bortezomib and ATRA against GFP-labeled human neuroblastoma xenografts

**Figure 6:**
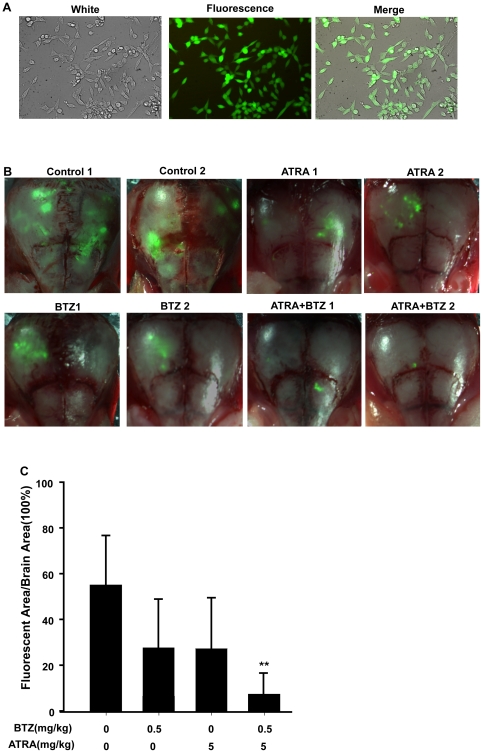
Combined antitumor activity of bortezomib and ATRA against GFP-labeled human neuroblastoma xenografts. A. Expression of GFP in CHP126 cells. B. Three days after orthotopic cell injection of CHP126-GFP, mice received 0.5 mg/kg BTZ i.v. and 5.0 mg/kg ATRA i.g. individually or in combination or saline solution (n = 2) every 3 d for 4 wk. C. GFP fluorescence in the brain and the respective area of the brain as determined by the Image-Pro plus 5.0 software. Each bar represents the mean ± SE. Statistical analysis was performed by t-test where appropriate. **P<0.01, significant difference from ATRA-treated animals. BTZ = bortezomib.

Based on our in vitro data and the proposed synergistic interaction between bortezomib and ATRA, we studied the combined antitumor activity of bortezomib and ATRA in vivo. GFP labeling in animal models is an extremely effective method for measuring cancer cell growth in target organs. The selected CHP126-GFP cells had a strikingly bright GFP fluorescence that remained stable in the absence of selective agents after numerous passages (Fig. 6A). CHP126-GFP cells were injected into the hippocampus of nude mice to mimic a highly aggressive pattern of human neuroblastoma. In previous reports, the maximal tolerated dose of bortezomib (1.0 mg/kg) was found to significantly inhibit tumor growth in several human xenograft models, including NB [30]. ATRA is usually administered orally at high dosages (10.0–50.0 mg/kg). Therefore, we initially treated mice with 0.5 mg/kg bortezomib and 5 mg/kg ATRA delivered in combination or as single agents twice a week for 4 wk. Fluorescence analysis was performed on brain sections derived from orthotopic tumor-bearing mice sacrificed 1 d after wk 4 of treatment. As shown in Fig. 6B and C, our data revealed that bortezomib plus ATRA significantly reduced NB cell proliferation compared with the individual drug treatments. The tumor growth inhibition rate of bortezomib (0.5 mg/kg) and ATRA (5.0 mg/kg) treatment was 84.7%, while the rates of bortezomib (0.5 mg/kg) or ATRA (5.0 mg/kg) alone were 43.4 or 47.1%, respectively (Table 1).

**Table 1:** Effects of ATRA and bortezomib on the growth of CHP126-GFP NB cells xenografts in nude mice.

Groups	Dose(mg/kg)	Fluoresce indensity (a.u.)	Inhibition Rate (%)
control	--	56.2±22.5	--
ATRA	5.0	29.1±24.4	47.1
bortezomib	0.5	31.8±25.3	43.4
ATRA+ bortezomib	5.0+0.5	8.6±7.1**	84.7

**: *P*<0.001,vs ATRA.

### Combined antitumor activity of bortezomib and ATRA against human neuroblastoma xenografts

**Figure 7:**
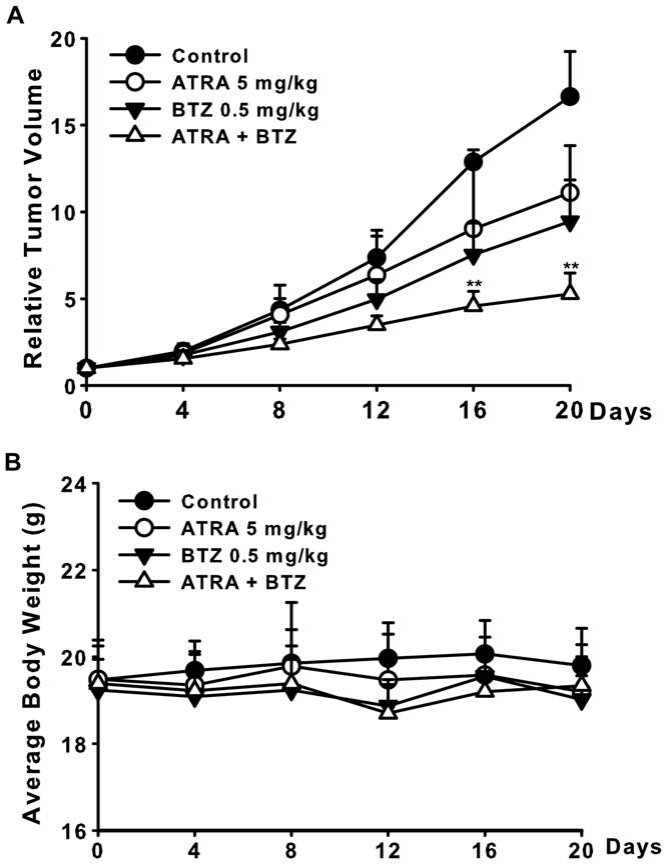
The synergistic effect of bortezomib and ATRA on CHP126 human xenograft models. The mice transplanted with CHP126 human xenograft were randomly divided into four groups and given injection of bortezomib, ATRA, combination or vehicle. A. Relative tumor volume. B. Average body weight. Data are expressed as the mean ± SE. Statistical analysis was performed by t-test where appropriate. **P<0.01, significant difference from ATRA-treated animals. BTZ = bortezomib.

For better determining synergistic interaction between bortezomib and ATRA in vivo, we studied the combined antitumor activity of bortezomib and ATRA in one more xenografts model (in mice bearing subcutaneous CHP126 neuroblastoma xenografts). The mice inoculated with CHP126 cells were divided into four groups (six mice per group); The i.p. administration of bortezomib at the dose of 0.5 mg/kg every 4 days for 20 days reduced 40.8% tumor growth; the i.g. administration of ATRA at the dose of 5 mg/kg every 2 days for 20 days resulted in a moderate growth inhibition of 27.9%; and the simultaneous treatment with a bortezomib and ATRA signiflcantly decreased the tumor growth by 56.8% (Table 2). As indicated in Fig. 7A, similar tumor growth inhibitory effect of bortezomib and ATRA were achieved on CHP126 xenograft models: with the dosage of 0.5 mg/kg, the T/C value of bortezomib -treated group was 54.0%; while the T/C value of 5 mg/kg ATRA-treated group is 68.9%. As expected, bortezomib plus ATRA exhibited distinct tumor growth inhibition (T/C value: 37.4%), with a signiflcant greater extent than bortezomib - or ATRA-treated mice (RTV of bortezomib + ATRA group vs. RTV of ATRA group: p<0.01). Toxicity in terms of progressive weight loss was not observed in any of the groups in the animalstudies (Fig. 7B).

**Table 2:** Combined antitumor activity of ATRA and bortezomib against human neuroblastoma cancer xenografts.

Groups	No. of animalsStart End	RTV	T/C%	Inhibition Rate (%)
control	6 6	16.6±2.6		--
ATRA 5 mg/kg	6 6	11.1±2.7	68.9	27.9
Bortezomib 0.5 mg/kg	6 6	9.4±2.4	54.0	40.8
ATRA+ bortezomib	6 6	5.3±1.1**	37.4	56.8

**: *P*<0.001,vs ATRA.

### The bortezomib and ATRA combined therapy showed little neurotoxicity in primary neurons

**Figure 8:**
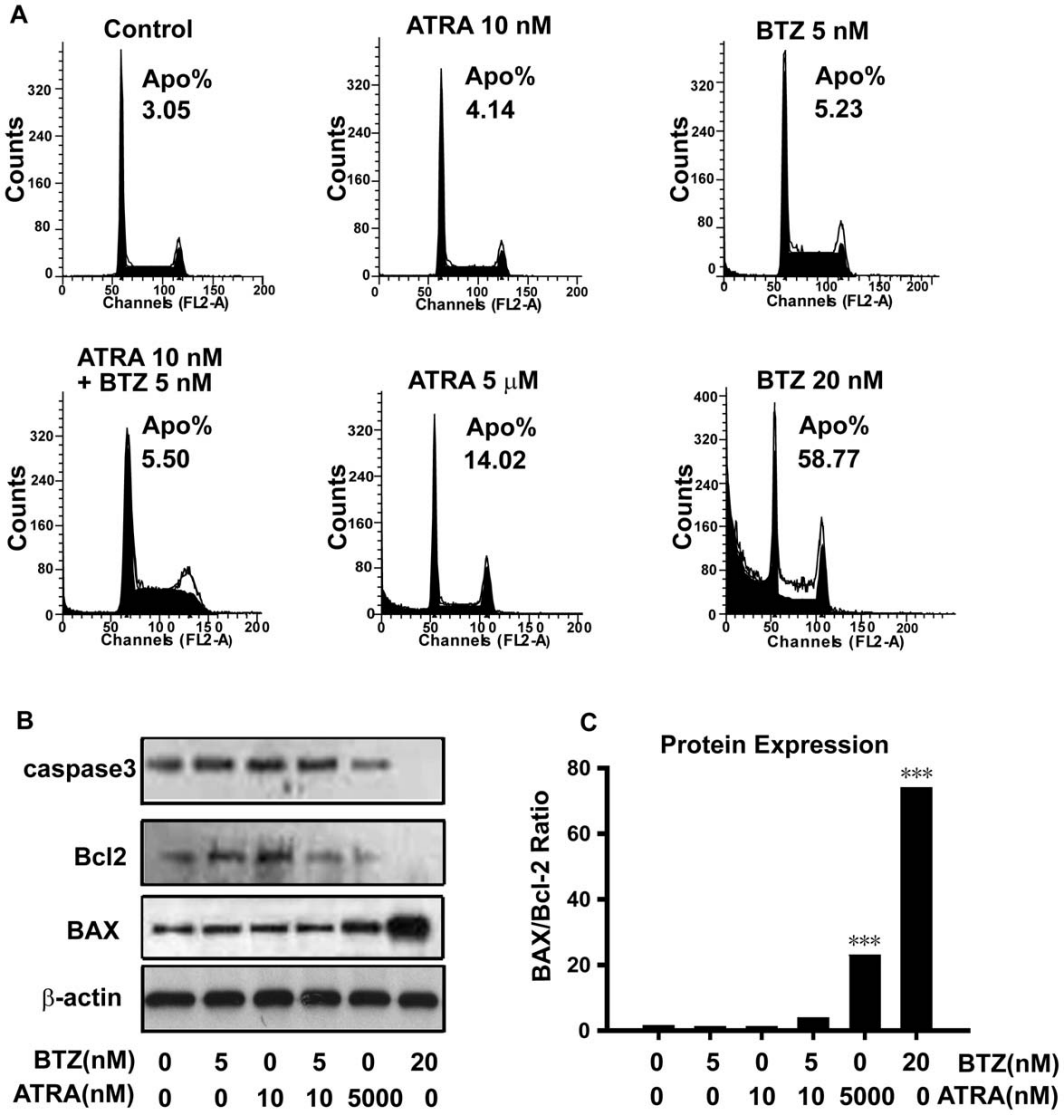
The bortezomib and ATRA combined therapy showed little neurotoxicity in primary neurons. A. Cells were incubated with 5.0 µM ATRA, 20.0 nM bortezomib, 5.0 nM bortezomib, 10.0 nM ATRA and 5.0 nM bortezomib plus 10.0 nM ATRA, as well as under control conditions, for 72 h. After treatment, cells were harvested and stained with PI. Flow cytometry was performed to quantify the percentages of apoptotic cells. B. Cells were incubated as above, and western blotting was used to detect the expression of caspase 3, Bax and Bcl-2. C. Ratios of Bax to Bcl-2 protein expression. Statistical analysis was performed by t-test where appropriate. ***P<0.001, significant difference from control cells. BTZ = bortezomib.

Bortezomib with ATRA showed strong synergistic neuronal differentiation in vitro and in vivo; however, we wondered whether this combination therapy enhanced neurotoxicity. Primary cerebellar granule neurons, which were isolated and cultured in our lab, were used to test the neurotoxicity of the combination therapy. Our results indicated that ATRA (10.0 nM) or bortezomib (5.0 nM) treatment both alone and in combination caused little or no decrease in cell viability in primary rat neurons as measured by the MTT assay (Table 3). On the other hand, ATRA (5.0 µM) or bortezomib (20.0 nM) treatment (3,30) of NB cells induced 24.3% or 67.6% neuroblastoma cell cytotoxicity (shown in Table 1), which was consistent with previous reports. Consistent with the cell viability results, PI staining also showed that ATRA (10.0 nM) or bortezomib (5.0 nM), alone or in combination caused little cell apoptosis in primary rat neurons. However, as shown in Fig. 8A, about 14.02% and 58.77% of cells were apoptotic (sub-G1) after ATRA (5.0 µM) or bortezomib (20.0 nM) treatment. To further elucidate the mechanism of the decreased neuronal toxicity of the combination therapy, expression of the apoptosis related protein caspase 3 was examined by western blot analysis. Treatment with high concentrations of either bortezomib (20.0 nM) or ATRA (5.0 µM) caused decreased the expression of caspase 3 (Fig. 8B), whereas, treatment with lower concentrations of bortezomib (5.0 nM) and ATRA (10.0 nM) alone or in combination remained the same. Because the MTT assay is also used to assess mitochondrial dysfunction in neurons, we examined the expression of mitochondria-related proteins (Bax and Bcl-2). Expression of the pro-apoptotic protein Bax was upregulated by treatment with high concentrations of bortezomib or ATRA, while low concentrations of the combination therapy did not upregulate Bax. At the same time, the expression level of the anti-apoptotic protein Bcl-2 in primary neurons remained the same as cells treated with bortezomib (5.0 nM) and ATRA (10.0 nM). However, Bcl-2 expression decreased after treatment with high concentrations of bortezomib or ATRA (Fig. 8B and C). Taken together, these results revealed that bortezomib (5.0 nM) combined with ATRA (10.0 nM) had little neuronal toxicity, which was partially associated with the inactivation of apoptosis. Using the concentrations of bortezomib and ATRA previously reported [15], [31] for NB treatment, we showed that high concentrations of these drugs induced disruption of the mitochondrial potential gradient that resulted in neuronal apoptosis.

**Table 3:** Effects of ATRA and bortezomib on the survive of primary rat neuron cells.

Groups	Concentration (nM)	Inhibition Rate (%)
Control	--	--
ATRA	10.0	9.2
bortezomib	5.0	11.7
ATRA+ bortezomib	10.0+5.0	14.5
ATRA	5000.0	24.3*
bortezomib	20.0	67.6 ***

*: *P*<0.05, ***: *P*<0.001,vs control.

## Discussion

There are numerous obstacles in the development of therapies for pediatric cancer. The rarity of these diseases is a considerable problem. NB is the most common extracranial pediatric solid tumor, yet only 800 children and adolescents are diagnosed each year in the United States [32]. In sharp contrast, the most common adult solid tumor, lung cancer, is diagnosed in more than 1 million new cases annually [33]. Because few people are affected by NB, there is reduced market incentive for industry-based drug development. Only a limited number of drugs can be tested, so drug combination therapies have played a prominent role in neuroblastoma treatment.

The concept of “differentiation therapy” has gained considerable interest in recent years. Despite both in vitro and clinical trial data suggesting that ATRA-induced differentiation could be an alternative therapeutic approach for neuroblastoma, the use of ATRA is limited by a number of problems, including serious systemic toxicity and the generation of resistance [1]–[3]. Thus, the search for potent enhancers of ATRA-induced differentiation in neuroblastoma cells is a desirable therapeutic goal.

The present study showed that bortezomib significantly enhanced the ATRA-induced differentiation of neuroblastoma cells. The combination of 5 nM bortezomib with 10 nM ATRA induced cell differentiation and was equivalent to that obtained with 5 µM ATRA alone, indicating that the addition of bortezomib could lower the concentration of ATRA by 500-fold. This reduction in the ATRA dosage could mitigate its side effects. At the same time, the concentration of bortezomib used in the combination therapy was 5 nM, which is 4-fold lower compared with the currently acceptable concentration used for the treatment of NB. This concentration reduction could mitigate its neuronal toxicity. Therefore, bortezomib could effectively promote the ATRA-induced terminal differentiation of neuroblastoma.

In light of the synergistic interaction between bortezomib and ATRA and our assumption that the lower concentrations could alleviate the side effects of these two drugs, we used primary cultured rat neurons to analyze the effect of the combination therapy on neuronal toxicity. We showed that 5.0 nM bortezomib combined with 10.0 nM ATRA had little effect on primary neurons, while 20.0 nM bortezomib or 5.0 µM ATRA alone caused remarkable neuronal apoptosis. Mitochondria are central to the apoptosis activation pathway in many physiological and pathological conditions. Members of the Bcl-2 family of proteins are known to affect mitochondrial function and regulate the release of apoptosis-activating factors [34]. Caspase 3 plays a central role in the execution of the apoptotic program and can also be activated by Bcl-2 family proteins [35]. In our study, a decrease in Bcl-2 and an increase in Bax protein expression levels were observed in primary cultured neurons treated with high concentrations of bortezomib or ATRA; therefore, the ratio of Bax to Bcl-2 was altered in favor of apoptosis. In addition to the increase in the Bax/Bcl-2 ratio, we observed the activation of caspase 3. Altogether, compared to 5.0 µM ATRA and 20.0 nM bortezomib alone, the combination of bortezomib and ATRA had no effect on the mitochondrial membrane potential gradient, which leads to little neuronal toxicity.

RA plays an important role in the function of the adult brain, which has been shown to synthesize RA and express retinoid receptors. Retinoic acid receptors (RARs: α, β and γ) and retinoid X receptors (RXRs: α, β and γ) are structurally related members of the steroid hormone receptor family of ligand-dependent transcription factors [3], [4]. Human NB cells express RAR isoforms in response to retinoic acid treatment; however, the expression levels varied among the different isoforms. In NB cell lines, the basal level of RARα expression was high and that of RARβ was extremely low. The expression of RARα in NB is directly controlled by the RAR/retinoic acid signaling pathway [11]. A previous study showed that RARα expression was ubiquitin-dependent and decreased during ATRA-induced neuronal differentiation, which possibly makes cancer cells insensitive to ATRA [12]. In our study, bortezomib blocked the reduction in RARα expression with ATRA treatment. RARβ has been suggested to play an important role in the biological functions of RA and to be associated with cellular sensitivity to retinoids in different types of cancers. RARβ may act as a tumor suppressor, and there is evidence that RARβ induction by retinoids is important for inhibiting tumor cell growth. Although most malignant cells have a very low level of RARβ expression, retinoid-sensitive cancer cells are characterized by a marked induction in endogenous RARβ expression after retinoid treatment in vitro [26]. It is known that bortezomib enhances ATRA-induced RARβ expression. Therefore, bortezomib might influence ATRA-mediated retinoid receptor expression, which is one possible way that bortezomib synergistically increases ATRA-induced cell differentiation.

Cell differentiation-inducing agents trigger an intracellular signaling cascade, which leads to activation of mitogen-activated protein (MAP) kinases. Previous studies have suggested that the ERK/MAPK pathway is crucial for NGF-induced neuronal differentiation of cells because blocking ERK/MAPK activation inhibits neurite induction, and constitutive activation of the ERK/MAPK pathway results in neurite outgrowth [36]. However, other findings demonstrated that sustained activation of ERK/MAPK did not induce neurite outgrowth in dorsal root ganglionic (DRG) sensory and sympathetic neurons in SH-SY5Y cells [37]. Other studies have suggested that activation of the c-Jun amino-terminal kinase was required for RA-induced neuronal differentiation of P19 embryonal carcinoma cells. The sustained activation of p38 promoted neuronal differentiation, and inhibition of p38 by a specific inhibitor SB203580 or by expression of dominant-negative constructs of the p38 pathway blocked neuritis outgrowth in PC12 cells [38]. We found that the combination treatment failed to produce a further increase in ERK1/2 and p38 expression, but upregulation of JNK levels by bortezomib was further increased in the presence of ATRA. To address the role of JNK in bortezomib-enhanced neuronal differentiation induced by ATRA, we used the inhibitor SP600125 to block JNK activity. Treatment with SP600125 did not increase ATRA-induced neuronal differentiation in CHP126 cells treated with bortezomib and ATRA. These observations suggested that the activation of the JNK signaling pathway is obligatory for bortezomib and ATRA-induced neuronal differentiation.

p21 was initially isolated as a p53 responsive gene and characterized as a protein that was closely correlated with induced differentiation in multiple cell culture model systems. Increased p21 expression is responsible for cell cycle arrest during NGF-induced neuronal differentiation of PC12 cells. During the course of NB cell differentiation, p21 also plays important roles in regulating the cell cycle. In several NB cell lines, the expression of p21 was increased during ATRA-induced differentiation [39]. Herein, bortezomib further increased p21 expression, which was not regulated by ATRA. We considered the possibility that p21 might mediate these responses. To test this hypothesis, p21 expression was silenced in NB cells by p21 shRNA. We compared bortezomib and ATRA-induced neuronal differentiation in control and p21-silenced NB cells. There were no differences, suggesting that p21 is dispensable for bortezomib and ATRA-induced neuronal differentiation in NB cells.

Because a strong synergistic effect was also achieved by the co-administration of ATRA and bortezomib in vitro, we developed GFP-expressing neuroblastoma cells (CHP126-GFP). This provided a more sensitive and powerful means with which to fully visualize and examine the synergistic anticancer effect of ATRA and bortezomib in vivo. Meanwhile to better determine the synergistic effect in vivo, we studied the combined antitumor activity of bortezomib and ATRA in mice bearing subcutaneous CHP126 neuroblastoma xenografts. It showed that bortezomib (0.5 mg/kg) and ATRA (5.0 mg/kg) reduced neuroblastoma growth in vivo when the combination of drugs was delivered to implanted neuroblastomas in mice. It is noteworthy that neither bortezomib nor ATRA doses used in the in vivo experiments were lower than those currently used in preclinical trials. This combination therapy ameliorated the antineoplastic effects of ATRA or bortezomib without increasing or altering systemic toxicity.

To summarize, our present findings demonstrate synergistic efficacy of bortezomib and ATRA in neuroblastoma in vitro and in vivo. Bortezomib significantly improved the ATRA-induced anticancer activities, as shown by the synergistic inhibitory effects on cancer cell proliferation, the sensitized execution of differentiation. Bortezomib combined with ATRA reduced neuronal toxicity at high drug concentrations without affecting the inherent anticancer properties. These synergistic effects can probably be attributed, at least partially, to the upregulation of JNK. Collectively, the current preclinical study forms the critical basis for the development of an anticancer regimen consisting of a combination of bortezomib and ATRA.
